# USING SIMULATION IN DEMENTIA CAREGIVER RESEARCH: LESSONS LEARNED

**DOI:** 10.1093/geroni/igae098.3195

**Published:** 2024-12-31

**Authors:** Cheryl Lee, Rita Jablonski

**Affiliations:** University of Alabama at Birmingham, Birmingham, Alabama, United States; University of Alabama at Birmingham, Birmingham, Alabama, United States

## Abstract

The purpose of this methods presentation is to describe how simulation-based training was used to impart critical skills for two members of a dementia caregiver research team: the interventionist and the person monitoring treatment fidelity. To have sufficient rigor and reproducibility in a randomized clinical trial, highly detailed protocols and training procedures must be developed and consistently followed. Inadequate training of interventionists may result in delivery variations of the same intervention, negatively affecting outcomes and the study’s generalizability. Simulations followed the Healthcare Simulation Standards of Best Practice’s general criteria for prebriefing and debriefing. Four pre-licensure nursing students role-played as simulated family caregivers who faced care-resistant behaviors by persons living with dementia. They co-created their simulated identities with the principal investigator. A treatment fidelity checklist was created to ensure the interventionist adhered to the key components of the intervention protocol. Inter-rater reliability for the treatment fidelity checklist was 100%. Treatment fidelity scores for the interventionist ranged from 23.1% to 52.9%, indicating poor adherence to the intervention protocol. Debriefings with the interventionist revealed that the simulated caregivers were passive during the simulations and that she had difficulty suspending disbelief during the simulations, which contributed to poor adherence. Students shared during their debriefing that their simulated identities should have had more details. Simulation training was continued using two family caregivers with better treatment fidelity results (90%-95%). Simulation is a viable method for training research personnel, but care is needed to select actors with experiences that mirror the research conditions.

